# Predictive factors for the presence of invasive components in patients diagnosed with ductal carcinoma in situ based on preoperative biopsy

**DOI:** 10.1186/s12885-019-6417-3

**Published:** 2019-12-10

**Authors:** Kwan Ho Lee, Jeong Woo Han, Eun Young Kim, Ji Sup Yun, Yong Lai Park, Chan Heun Park

**Affiliations:** 1 Department of Surgery, Eunpyeong St. Mary’s Hospital, College of Medicine, The Catholic University of Korea, Seoul, South Korea; 2 Department of Surgery, Kangbuk Samsung Hospital, Sungkyunkwan University School of Medicine, 29 Saemunan-ro, Jongno-gu, Seoul, Seoul 03181 South Korea

**Keywords:** Ductal carcinoma in situ, Magnetic resonance imaging, Breast, Biomarkers

## Abstract

**Background:**

In patients diagnosed with ductal carcinoma in situ (DCIS) with needle biopsy before surgery, invasive component (IC) is often found in the postoperative tissue, which results in altered post-surgical care. However, there are no clinically available factors to predict IC, and few MRI studies are available for the detection of IC in DCIS patients. The purpose of this study was to evaluate which risk factors can predict IC preoperatively.

**Methods:**

Patients with a DCIS diagnosis based on preoperative biopsy, who underwent breast surgery Kangbuk Samsung Hospital between Jan 2005 and June 2018, were retrospectively evaluated. Clinico-pathological and breast MRI factors were compared between DCIS and DCIS with IC in postsurgical specimens.

**Results:**

Of the 431 patients with a preoperative diagnosis of DCIS, 34 (7.9%) showed IC during the postoperative pathological investigations, and 217 (50.3%) underwent breast MRI. Among MRI-related factors, Mass-like enhancement on MRI was the sole but significant predictor of IC (HR = 0.26, C.I. = 0.07–0.93, *p* = 0.038), while nipple-areolar complex invasion, enhancement peak and pattern were not statistically significant. Nuclear grade was the only significant predictor of IC in the analysis of other clinico-pathological factors (HR = 2.39, C.I. = 1.05–5.42, *p* = 0.038 in univariate analysis, HR = 2.86, C.I. = 1.14–7.14, *p* = 0.025 in multivariate analysis).

**Conclusions:**

Mass-like enhancement on MRI and high nuclear grade were associated with IC in patients with preoperative diagnosis of DCIS. Considering the high sensitivity of breast MRI for IC, further evaluation of the predictive value of MRI in preoperative DCIS patients is desirable.

## Background

With more than 2 million new cases worldwide in 2018, breast cancer is the most common cancer in women and the second most common cancer overall, according to World Cancer Research Fund International. In ductal carcinoma in situ (DCIS), cancer cells are limited to the duct, as indicated by the term ‘in situ’ or ‘in its original place’. Thus, theoretically, a sentinel lymph node biopsy (SLNB) is not essential for patients with DCIS, which does not invade adjacent tissues, lymph nodes, and organs beyond the basal cell layer in the mammary duct.

In practice, however, pathological diagnosis using core needle biopsy (CNB) or vacuum-assisted core biopsy (VACB) cannot completely exclude invasive component (IC) before surgery [1–3]. The presence of IC in surgical specimens results in upstaging of DCIS to invasive carcinoma, and changing the post-surgical care plan. It seriously degrades patients’ quality of life due to a reoperation for evaluation of axillary lymph node. Especially, when breast reconstruction is performed together, it may result in delayed adjuvant chemotherapy or radiotherapy due to complications of the reconstructed site. Also, Damaging the reconstructed site could be occurred due to radiotherapy or systemic treatment [4].

The purpose of this study was to evaluate which risk factors, including Magnetic resonance imaging (MRI), can predict IC preoperatively in patients diagnosed with DCIS. Previous studies that attempted to determine the risk factors or predictors for preoperative detection of IC yielded discordant results. Until now, only a few small studies evaluated the MRI features as clinical risk factors to predict IC prior to surgery [5–11].

## Methods

### Patients

We retrospectively evaluated the data of 439 patients diagnosed with DCIS based on the pathological analysis of preoperative biopsy specimens of patients who underwent breast surgery at Sungkyunkwan University, Kangbuk Samsung Hospital between Jan 2005 and June 2018. Patients without comprehensive clinico-pathologic data were excluded. Ultimately, 431 patients were eligible for analysis and retrospective review. Clinical information was extracted from medical records including age, imaging findings, biopsy method, tumor grade, pathology results after surgery, treatment information (surgery, radiotherapy, and chemotherapy) and status of estrogen receptor (ER), progesterone receptor (PR), and human epithelial growth factor receptor 2 (HER-2) expression. An MRI study was performed in 217 patients, and MRI-related factors including tumor size, nipple-areolar complex (NAC) status, enhancement pattern, dynamic curves, and enhancement peak were also gathered. This study was approved by the Institutional Review Board of Kangbuk Samsung Hospital, the Sungkyunkwan University of Korea, on March 3, 2017 (KBSMC 2017–03-018).

### Imaging-guided biopsy techniques

Ultrasonography (US) was performed using a high-resolution 5–12 MHz linear array transducer in CNB. In the examination, patients’ breasts were examined in transverse and radial planes with both arms over the head. We obtained bilateral mammograms (medio-lateral oblique and cranio-caudal views) using a standard mammographic unit (Mammomat 3000; Nova Siemens). A specimen detected on US was obtained via US-guided CNB using 16- or 18-gauge needle, and the lesion detected only on mammography was obtained via stereotactic core needle biopsy or excisional biopsy with needle localization. We used an automated gun and a 14-gauge dual-action semiautomatic core biopsy needle with a 22-mm-throw.

### Magnetic resonance imaging and protocol

Bilateral breast MRI was performed with a 3.0-Tesla system (Achieva; Philips Medical System, Best, the Netherlands). Images were obtained with the following protocol: an axial turbo spin-echo T2-weighted(W) imaging sequence with a repetition time [TR]/echo time [TE], 3790/100; matrix size, 332× 316; field of view [FOV], 200× 340 mm; section thickness, 3 mm; gap, 1 mm, T1-W with a TR/TE, 620/10; matrix size, 332 × 332; FOV, 200× 340 mm; section thickness, 3 mm; gap, 1 mm, and dynamic contrast-enhanced examination using a fat-suppressed T1-W 3D fast field echo sequence with a TR/TE, 7.0/3.5; matrix size, 452× 410; FOV, 340× 340 mm; section thickness, 2 mm; no gap. For the evaluation of the axilla, delayed axial spin-echo T1-W images with a TR/ TE, 532/10; matrix size, 448× 378; FOV, 380× 380 mm; section thickness, 5 mm; gap, 2.5 mm were obtained using a body coil. Dynamic images were acquired before and six times after intravenous injection of 1.0 M gadobutrol (7.5 mL, Gadovist; Bayer Schering Pharma, Berlin, Germany). Image acquisition time per one dynamic scan was 60–120 s. MRI reports were interpreted by two radiologists with 5–15 years’ experience in breast imaging (S.H.K., S.H.C) according to the American College of Radiology Breast Imaging Reporting and Data System (BI-RADS) [12]. Lesion size, lesion characteristics (mass or non-mass), lesion morphology (shape, margin, and internal enhancement in mass lesion), and time-signal intensity curve patterns (fast, medium, or slow/persistent, plateau, or washout) were determined.

### Pathologic evaluation and immunohistochemical (IHC) analyses

All patient samples were obtained via image-guided needle biopsy (CNB or VACB) or surgical specimens were fixed in 10% formaldehyde solution and then embedded in paraffin. A section was stained with hematoxylin and eosin for determination of tumor size, histologic type, nuclear grade, margin status, and presence of metastatic lymph nodes. IHC analysis was performed for the evaluation of ER, PR. HER2 status and ki-67 by an experienced breast histopathologist.

### Statistical analyses

All statistical analyses were performed using the R version 3.3.2 [13, 14]. Fisher’s exact test or the Chi-square test for categorical variables and Student’s *t*-test for continuous variables were used to compare the continuous variables between patients with DCIS and those with IC. The Cox proportional hazards regression model was used for univariate and multivariate analyses. All tests were two-sided and *p* <  0.05 was considered statistically significant.

## Results

The baseline characteristics of 431 patients are listed in Table 1. These characteristics were compared between patients diagnoses with DCIS and those carrying IC. In 431 patients in our hospital diagnosed with DCIS before surgery, 397 (92.1%) were reported having DCIS and 34 patients (7.9%) had DCIS with IC based on pathology findings post-surgery. The characteristics were compared between patients diagnoses with DCIS and those with IC. Factors such as biopsy method (needle biopsy or excisional biopsy, *p* = 0.001), surgery type (breast-conserving surgery or total mastectomy, *p* = 0.009), pathologic DCIS size (≤ 2 cm, > 2 cm, or no residual DCIS, *p* = 0.010) varied significantly between the two groups.

**Table 1:** Clinico-pathological features of 431 patients with a preoperative histopathologic diagnosis of DCIS

	DCIS (*N* = 397)	Invasive component (*N* = 34)	*p*
Age (yrs)	50.2 ± 10.6	50.3 ± 11.4	0.967
US tumor size (mm, mean ± SD)			0.663
≤ 2 cm	251 (63.2%)	19 (55.9%)	
> 2 cm	133 (33.5%)	14 (41.2%)	
Not seen	13 (3.3%)	1 (2.9%)	
US finding			0.567
Non-mass	42 (10.6%)	2 (5.9%)	
Mass	355 (89.4%)	32 (94.1%)	
Mammographic finding			0.286
Asymmetry	25 (6.3%)	2 (5.9%)	
Microcalcification	292 (73.6%)	21 (61.8%)	
Architecture distorsion	8 (2.0%)	2 (5.9%)	
No specific finding	72 (18.1%)	9 (26.5%)	
Biopsy methods			0.001
Needle Biopsy	250 (63.0%)	32 (94.1%)	
Excisional Biopsy	147 (37.0%)	2 (5.9%)	
Surgery type			0.009
Breast conserving surgery	204 (51.4%)	9 (26.5%)	
Total mastectomy	193 (48.6%)	25 (73.5%)	
Pathologic DCIS size			0.010
≤ 2 cm	241 (60.7%)	12 (35.3%)	
> 2 cm	148 (37.3%)	20 (58.8%)	
No residual DCIS	8 (2.0%)	2 (5.9%)	
Invasive tumor size (mm, mean ± SD)		7.0 ± 8.9	< 0.001
pN			0.117
0	393 (99.0%)	32 (94.1%)	
1	4 (1.0%)	2 (5.9%)	
Multifocality			1.000
Absent	313 (78.8%)	27 (79.4%)	
Present	84 (21.2%)	7 (20.6%)	
Nuclear grade			0.125
Low/intermediate	319 (80.4%)	23 (67.6%)	
High	78 (19.6%)	11 (32.4%)	
Estrogen receptor			0.177
-	88 (22.2%)	12 (35.3%)	
+	302 (76.1%)	22 (64.7%)	
Unknown	7 (1.8%)	0 (0%)	
Progesterone receptor			0.130
-	123 (31.0%)	16 (47.1%)	
+	267 (7.3%)	18 (52.9%)	
Unknown	7 (1.8%)	0 (0%)	
HER2			0.679
-	241 (61.2%)	20 (58.8%)	
Overexpression	147 (37.0%)	14 (41.2%)	
Unknown	7 (1.8%)	0 (0%)	
Ki-67(%)	8.8 ± 9.1	15.3 ± 25.5	0.271
Closest margin (mm)	4.8 ± 5.2	5.1 ± 4.3	0.788
Follow up periods (months)	46.0 ± 35.2	61.6 ± 46.5	0.064
Any recurrence			1.000
Absent	389 (98.0%)	33 (97.1%)	
Present	8 (2.0%)	1 (2.9%)	
Chemotherapy			< 0.001
no	381 (96.0%)	18 (52.9%)	
yes	16 (4.0%)	16 (47.1%)	
Hormone therapy			0.147
no	90 (22.7%)	12 (35.3%)	
yes	307 (77.3%)	22 (64.7%)	

Data are expressed as means±standard deviations or n (%)

*Abbreviations*: *DCIS* Ductal carcinoma in situ, *US* Ultrasonography, *HER2* Human epidermal growth factor receptor 2

MRI scan was performed on 217 of 431 patients and the differences between the two groups are listed in Table 2. Non-mass enhancement (NME) was more common finding in the DCIS group than IC group (*p* = 0.002). There were no statistically significant differences in MRI tumor size (≤ 2 cm, > 2 cm, or not seen, *p* = 0.172), nipple-areolar complex invasion (absent or present, *p* = 1.000), enlarged lymph node (absent or present, *p* = 0.612), enhancement peak (≤ 100% or > 100%, *p* = 0.649), initial enhancement pattern of dynamic curve (rapid, medium, or slow, *p* = 0.735), and kinetic pattern (persistent, plateau, or washout, *p* = 0.253).

**Table 2:** MRI findings of 217 patients with a preoperative histopathologic diagnosis of DCIS

	DCIS (*N* = 202)	Invasive component (*N* = 15)	*p*
MRI tumor size			0.172
≤ 2 cm	99 (49.0%)	6 (40.0%)	
> 2 cm	101 (50.0%)	8 (53.3%)	
Not seen	2 (1.0%)	1 (6.7%)	
Nipple-areolar complex invasion			1.000
Absent	191 (94.6%)	14 (93.3%)	
Present	11 (5.4%)	1 (6.7%)	
Enlarged lymph node			0.612
Absent	166 (82.2%)	11 (73.3%)	
Present	36 (17.8%)	4 (26.7%)	
MRI NME			0.002
Mass	102 (50.5%)	12 (80.0%)	
NME	100 (49.5%)	3 (20.0%)	
Enhancement peak			0.649
≤ 100%	37 (18.3%)	4 (26.7%)	
> 100%	165 (81.7%)	11 (73.3%)	
Initial enhancement pattern of dynamic curve			0.735
Rapid	162 (80.2%)	11 (73.3%)	
Medium	26 (12.9%)	3 (20.0%)	
Slow	14 (6.9%)	1 (6.7%)	
Kinetic pattern			0.253
Persistent	68 (33.7%)	4 (26.7%)	
Plateau	68 (33.7%)	3 (20.0%)	
Washout	66 (32.7%)	8 (53.3%)	

Data are expressed as n (%)

*Abbreviations DCIS* Ductal carcinoma in situ, *US* Ultrasonography, *NME* Non-mass enhancement

### Clinico-pathological predictors of invasive component in patients with preoperative needle biopsy-based diagnosis of DCIS

Among 431 patients diagnosed with DCIS via preoperative biopsy, 149 who were diagnosed with DCIS preoperatively by excisional biopsy, were excluded for this analysis. We investigated the clinico-pathological factors that predicted IC in the surgical specimen obtained during the operation. The results revealed that nuclear grade (hazard ratio (HR) = 2.39, confidence interval (C.I.) = 1.05–5.42, *p* = 0.038 in univariate analysis, HR = 2.86, C.I. = 1.14–7.14, *p* = 0.025 in multivariate analysis) was the only significant risk factor for IC in both univariate and multivariate analyses of clinico-pathological predictors (Table 3). Age, US tumor size, US finding, mammographic finding, multifocality, ER, PR, HER-2, Ki-67, and type of operation carried no significant value as predictors of IC.

**Table 3:** Clinico-pathological predictors of invasive component in patients with preoperative needle biopsy-based diagnosis of DCIS

Variable	Risk for invasive component			
	Univariate analysis		Multivariate analysis	
	HR (95% CI)	p	HR (95% CI)	*p*
Age (> 50)	0.75 (0.36–1.59)	0.458	0.82 (0.38–1.79)	0.623
US tumor size (> 2 cm)	1.31 (0.61–2.82)	0.489	1.40 (0.60–3.26)	0.441
US finding (mass)	1.52 (0.19–12.10)	0.693		
Mammographic finding (microcalcification)	0.73 (0.34–1.56)	0.416	0.83 (0.36–1.91)	0.666
Multifocality (present)	0.95 (0.39–2.31)	0.906		
Nuclear grade (high)	2.39 (1.05–5.42)	0.038	2.86 (1.14–7.14)	0.025
Estrogen receptor (positive)	0.66 (0.30–1.45)	0.305		
Progesterone receptor (positive)	0.66 (0.31–1.38)	0.266		
HER-2(overexpression)	1.05 (0.49–2.22)	0.904		
Ki-67 (> 14%)	1.32 (0.48–3.62)	0.589		
Type of operation (Mastectomy)	2.25 (1.00–5.05)	0.050	2.27 (0.94–5.50)	0.069

*Abbreviations DCIS* Ductal carcinoma in situ, *US* Ultrasonography, *HER2* Human epidermal growth factor receptor 2

In MRI-related factors, NME on MRI revealed as a sole significant clinical predictor of DCIS without IC (HR = 0.26, C.I. = 0.07–0.93, *p* = 0.038). Parameters such as MRI tumor size, nipple-areolar complex invasion, enlarged lymph node, enhancement peak, initial enhancement pattern of dynamic curve, and kinetic pattern were not significant predictors of IC (Table 4).

**Table 4:** Clinical predictors of invasive component on MRI in patients with preoperative diagnosis of DCIS

Variables	Risk for invasive component	
	HR (95% CI)	*p*
MRI tumor size (> 2 cm)	1.31 (0.44–3.90)	0.632
Nipple-areolar complex invasion (present)	1.24 (0.15–10.31)	0.842
Enlarged lymph node (present)	1.68 (0.51–5.57)	0.399
MRI NME (NME)	0.26 (0.07–0.93)	0.038
Enhancement peak (> 100%)	0.62 (0.19–2.04)	0.429
Initial enhancement pattern of dynamic curve (rapid)	1.47 (0.45–4.87)	0.526
Kinetic pattern (wash-out)	2.35 (0.82–6.77)	0.112

*Abbreviations DCIS* Ductal carcinoma in situ, *NME* Non-mass enhancement

## Discussion

In our study, we evaluated factors related to upstaging of the diagnosis of breast cancer from pre-operative DCIS to post-operative invasive carcinoma. In previous studies, the risk factors associated with this upstaging such as young age, palpability, nipple discharge, specimen number, extent of microcalcification, mass size, high nuclear grade, axillary lymph node involvement, multicentric lesion, contralateral lesion, and the presence of HER-2 over expression, were reported as predictors of DCIS with IC prior to surgery [1, 4–11, 15–28]. However, these studies were small in sample size and reported inconsistent results, and therefore, no predictable factors for IC were available clinically. In particular, few studies related to MRI, and the number of subjects in these studies was very small. By contrast, the scale of our research is relatively large.

There has been no consensus on age as a predictor for DCIS with IC. Yen et al. found that 55 years of age or younger was the predictor for IC in DCIS [16]. However, Nori et al. suggested that age greater than 55 years at diagnosis was significantly associated with a higher risk of IC [10]. Considering inconsistent results between studies and few studies suggesting age as a significant predictor, age is not likely a predictor, and thus, consistent with our results.

Mixed results suggest nuclear grade as a predictor across the studies. In our analysis, high grade was the only pathological predictor for IC (HR = 2.39, C.I. = 1.05–5.42, *p* = 0.038 in univariate analysis, HR = 2.86, C.I. = 1.14–7.14, *p* = 0.025 in multivariate analysis). Guillot et al. noted that high-grade DCIS was a risk factor of IC [4]. Brennan also reported that high-grade DCIS was significantly associated with a underestimation of DCIS [24]. However, several studies found no relationship between the high grade and the likelihood of upstaging [1, 16, 17, 19, 29]. This discrepancy is a reflection of the controversy excluding microinvasion from histologic grade; however, this pathological factor is still worthy of investigation in future studies along with other features such as comedo-necrosis.

A larger lesion size in imaging studies has been suggested a predictive factor for upstaging to invasive cancer. In mammography, the cutoff value of size ranged from 20 to 50 mm in previous studies [9, 27]. Lee et al. reported that sonographic lesions larger than 20 mm were significantly related to invasion [30]. Park et al. found that sonographic mass sizes larger than 32 mm were strong risk factors for DCIS with IC in conjunction with findings suggesting that mass sizes larger than 30 mm on an MRI were strong predictors for DCIS with IC [9]. Although we evaluated US and MRI tumor size as risk factors for occult invasion, no statistically significant data were obtained with these variables. These varying results might be attributed to insufficient case numbers, use of different cut-off values and diagnostic modalities in each study, and interobserver variability.

The role of MRI in screening and diagnosis of invasive breast cancer is well known [10], but few studies investigated MRI features as preoperative risk factors for occult IC in DCIS patients. Previously, Goto el al. reported that higher signal intensity of the enhancing lesion on fat-suppressed (FS) T2W image was a good predictor of IC [5]. Wisner et al. concluded that rapid early enhancement (*p* = 0.001) and washout kinetics correlated with occult IC in DCIS patients [8]. In contrast to these reports, Park pointed out that a lower signal intensity on FS T2WI MRI, and heterogeneous or rim enhancement on an MRI were significant variables, but not the higher signal intensity of the enhancing lesion on FS T2WI or the time-signal intensity curve pattern [9]. In our analysis, MRI-related variables such as enhancement peak, initial enhancement peak of dynamic curve, and kinetic pattern, showed no significant differences in IC detection. NME was the only predictive factor for DCIS without IC (HR = 0.26, C.I. = 0.07–0.93, *p* = 0.038).

Based on breast MRI, lesion types were classified into NME or mass. NME on an MRI has been investigated previously as a predictor of DCIS with IC. However, most of them showed no significant correlation between NME and occult invasion [5–11, 26, 28]. Only two studies suggested that mass-like enhancement was a predictor of DCIS with IC, which is consistent with our study [11, 22]. It may be explained by previous studies about non-mass lesion (NML) on breast US [31–35]. Most breast malignancies with NML on US were DCIS and only 5.6% of them were associated with IC alone [35]. Although our results were supported by larger case numbers than in previous studies, the lack of concordance between previous studies and our results suggests the need for re-evaluation of NME on a larger scale and for prospective studies to predict IC.

In kinetic curve assessments, no typical pattern differentiated invasive carcinoma from pure DCIS. Previously, rapid initial enhancement and the washout kinetic curve were reported as predictors of DCIS with IC by Wisner et al. [8]. Another study by Goto el al., showed that kinetic descriptors for initial and delayed phase enhancement had no significant role [5]. A few additional studies also evaluated this enhancement pattern; however, they revealed no significant results [9, 11]. Although no obvious conclusions can be drawn based on MRI enhancement patterns, considering the diagnostic value for breast malignancy, it appears that their significance as a predictor should be elucidated in additional trials in the future.

This study has some limitations. Our study was designed retrospectively, which may lead to bias between the group exposed to MRI scan and the unexposed group. In addition, the difference in tissue volume between CNB and VANB may have affected the diagnostic ability.

## Conclusion

We suggest that high nuclear grade and mass-like enhancement on MRI are predictors of IC in patients with a preoperative diagnosis of DCIS. In clinical practice, no factors are available to predict IC yet. Considering the high sensitivity of breast MRI for an IC, we expect further evaluation of the predictive value of MRI for IC in patients with preoperative DCIS. Therefore, a score system or a calculator that includes clinico-pathological factors and MRI factors is needed to predict DCIS with IC in the future, which can facilitate the treatment plan for patients with DCIS.

## Data Availability

The datasets used and/or analyzed during the current study are available from the corresponding author on reasonable request.

## Abbreviations

ADC: Apparent diffusion coefficient
CNB: Core needle biopsy
DCIS: Ductal carcinoma in situ
ER: Estrogen receptor
FS: Fat-suppressed
HER2: Human epidermal growth factor receptor 2
IC: Invasive component
IHC: Immunohistochemical
MRI: Magnetic resonance imaging
NAC: Nipple-areolar complex
NME: Non-mass enhancement
NML: Non-mass lesion
PR: Progesterone receptor
SLNB: Sentinel lymph node biopsy
US: Ultrasonography
VACB: Vacuum-assisted core biopsy
